# Hereditary Sensory and Autonomic Neuropathy 2B Caused by a Novel *RETREG1* Mutation (c.765dupT) and Paternal Uniparental Isodisomy of Chromosome 5

**DOI:** 10.3389/fgene.2019.01085

**Published:** 2019-10-31

**Authors:** Geun-Young Park, Dae-Hyun Jang, Dong-Woo Lee, Ja-Hyun Jang, Joungsu Joo

**Affiliations:** ^1^Department of Rehabilitation Medicine, College of Medicine, The Catholic University of Korea, Seoul, South Korea; ^2^Green Cross Genome, Yongin, South Korea; ^3^EONE-DIAGNOMICS Genome Center, Incheon, South Korea

**Keywords:** hereditary sensory and autonomic neuropathy, consanguineous marriage, frameshift mutation, uniparental isodisomy, homozygous

## Abstract

Hereditary sensory and autonomic neuropathy (HSAN) 2B is a rare disease and has been reported mostly in offspring of consanguineous parents. Here we report the case of a patient born to non-consanguineous parents who was diagnosed with HSAN 2B caused due to a novel frameshift mutation (NM_001034850.2: c.765dupT/p.Gly256TrpfsTer7) in the *RETREG1* gene and paternal uniparental isodisomy of chromosome 5. Uniparental isodisomy of chromosome 5 is also a rare condition, and these two rare events lead to homozygous expression of a recessive mutation, as in the present case. Clinicians should be aware that autosomal recessive disorders due to homozygous variants can occur because of uniparental disomy in offspring of non-consanguineous parents.

## Background

To date, a total of eight families related to hereditary sensory and autonomic neuropathy (HSAN) 2B due to *RETREG1* mutation have been reported since Kurth et al. (2009) first reported four such families (Davidson et al., 2012; Murphy et al., 2012; Ilgaz Aydinlar et al., 2014; Wakil et al., 2018). HSAN 2B is characterized by progressive sensory deficit and variable autonomic and motor involvement. Because few *RETREG1* mutations have been reported, there is no clear description of the phenotype of HSAN 2B.

Since the first report of uniparental disomy of chromosome 5 in 1994, several cases have been additionally reported (Brzustowicz et al., 1994; Seal et al., 2006; Lin et al., 2007; Garcia et al., 2014; Melone et al., 2014; Numata et al., 2014; Janecke et al., 2015; Wang et al., 2015). Uniparental disomy of chromosome 5 is also a rare condition with no clear description of clinical significance such as imprinting effects. Here, we describe a novel frameshift mutation (NM_001034850.2: c.765dupT/p.Gly256TrpfsTer7) in the *RETREG1* gene and paternal uniparental isodisomy of chromosome 5 in a patient diagnosed with HSAN 2B. Written informed consent was obtained from the patient for the publication of this case report after she had been briefed about the study.

## Case Presentation

**Figure 1:**
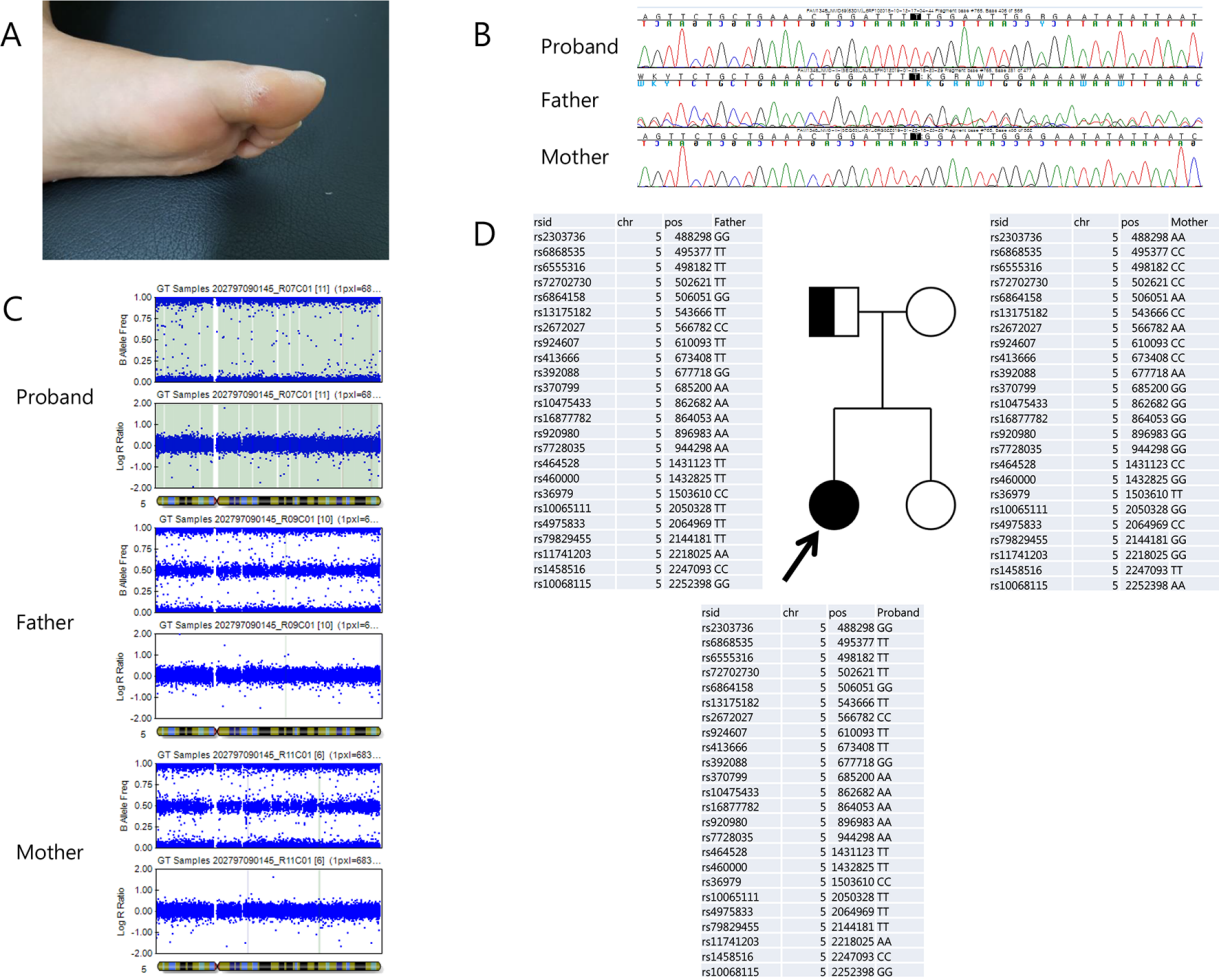
**(A)** Recently heeled foot ulcer. **(B)** Chromatograms of the reverse *RETREG1* gene sequence in the proband (top), her father (middle), and her mother (bottom). **(C)** Single nucleotide polymorphism array analysis of chromosome 5 from the proband (top), her father (middle), and her mother (bottom) showed uniparental isodisomy of chromosome 5 in the proband. **(D)** Pedigree: The filled box indicates the patient and the half-filled box indicates the father as the heterozygous carrier. Genotypes of chromosome 5 in the proband and her parents revealed that her uniparental isodisomy originated from the father.

The proband was a 22-year-old female who visited our clinic for evaluation of a sensory disturbance in both lower extremities and unstable gait. The patient was born to non-consanguineous parents in a Korean family with no familial history of hereditary disorders. She had normal birth history and developmental milestones. Her symptoms had started to appear at the age of approximately 10 years and gradually progressed. The patient exhibited a decreased sensitivity to pain, temperature, touch, and vibration and decreased sense of position in both lower extremities, with an ulcer on her left toe, which had lasted more than a year and had healed recently (**Figure 1A**). She had mild distal motor weakness in her lower extremities (Medical Research Council scale 4), spasticity, and increased deep tendon reflex in both lower extremities. She exhibited normal intelligence and no autonomic symptoms. A nerve conduction study indicated sensory axonal polyneuropathy more than motor, and sympathetic skin response was impaired on both the soles. The patient underwent genetic testing, using a targeted gene sequencing panel, analyzing 410 genes associated with genetic neuromuscular diseases. Genomic DNA was extracted from the peripheral blood of the patient. Library preparation and target enrichment were performed using the hybridization capture method. Custom oligo design and synthesis were done by Agilent (USA). Massively parallel sequencing was performed using 2 × 150 bp in the paired end mode of NextSeq platform (Illumina, San Diego, CA). A novel frameshift homozygous variation was detected in the patient’s *RETREG1* gene, c.765dupT/p.Gly256TrpfsTer7. However, of the patient’s parents, only the father was identified as a carrier of the variation in Sanger sequencing (**Figure 1B**). Furthermore, a single nucleotide polymorphism array (Infinium Global Screening Array-24+ v2.0, Illumina) was performed with DNA from the patient and her parents, revealing paternal uniparental isodisomy of chromosome 5 (**Figures 1C, D**).

## Discussion

Clinical features of the present case and all previously reported cases of HSAN 2B have been summarized in **Table 1**. HSAN 2B is a rare genetic disorder and has been mostly reported in consanguineous families, unlike our case. There have been six pathologic variants in the *RETREG* including the present case, which was the frameshift, nonsense, and splice site type. Furthermore, all reported patients were the homozygous genotype which suspicious of consanguineous family. All cases have similar characteristics including early onset (1st–2nd decade), progressive sensory disturbance, distal motor weakness, skin ulcer, and/or spasticity, with variable autonomic involvement. Although the pathomechanism through which *RETREG* mutation causes HSAN 2B was postulated to be the inhibition of turnover and degradation of the endoplasmic reticulum, it is largely unknown (Islam et al., 2018).

**Table 1:** Clinical features of reported cases with hereditary sensory and autonomic neuropathy 2B.

	Family 1	Family 2	Family 3	Family 4	Family 5	Family 6	Family 7	Family 8	Our case
Reference	Kurth et al. (2009)	Kurth et al. (2009)	Kurth et al. (2009)	Kurth et al. (2009)	Murphy et al. (2012)	Ilgaz Aydinlar et al. (2014)	Wakil et al. (2018)	Wakil et al. (2018)	Park et al. (2019)
Variants	c.926C > G(p.Ser309Ter)	c.18_19delTC(p.Pro7GlyfsTer133)	c.433C > T(p.Gln145Ter)	c.873+2T > C	c.433C > T(p.Gln145Ter)	c.826delA(p.Ser276ValfsTer8)	c.926C > G(p.Ser309Ter)	c.926C > G(p.Ser309Ter)	c.765dupT(p.Gly256TrpfsTer7)
Genotype	Homozygote	Homozygote	Homozygote	Homozygote	Homozygote	Homozygote	Homozygote	Homozygote	Homozygote
Origin	Saudi-Arabia	Turkey	Italy	Dubai	Somalian	Turkey	Saudi-Arabia	Saudi-Arabia	Korean
Number of affected patients	4	1	2	2	2	6	3	1	1
Consanguineous family	Yes	Not reported, from the same village	Not reported	Yes	Yes	Yes	Yes	Yes	No
Onset	1^st^ – 2^nd^ decade	1^st^ decade	1^st^ decade	2^nd^ decade	1^st^ decade	1^st^ decade	1^st^ decade	1^st^ decade	1^st^ – 2^nd^ decade
Sensory disturbance	Yes	Yes	Yes	Yes	Yes	Yes	Yes	Yes	Yes
Autonomic symptom	Yes	Yes	Yes	Not reported	No	Yes	Not reported	Not reported	No
Motor weakness	Not reported	Not reported	Not reported	Yes	Yes	Yes	Yes	Yes	Yes
Spasticity	Not reported	Not reported	Yes	Not reported	No	Yes	Yes	Yes	Yes
Ulcerations	Yes	Yes	Yes	Yes	Yes	Yes	Yes	Yes	Yes
Intelligence	Not reported	Not reported	Not reported	Not reported	Normal	Not reported	Normal	Not reported	Normal
Osteomyelitis	Yes	Yes	Yes	Yes	Yes	Not reported	Yes	Yes	No
Nerve conduction study	Sensory axonal	Sensory-motor axonal	Sensory-motor axonal	Sensory-motor axonal	Sensory-motor axonal	Sensory-motor axonal	Sensory axonal	Normal	Sensory-motor axonal

In addition, uniparental disomy of chromosome 5 has been rarely reported, with approximately 15 cases reported to date (http://upd-tl.com/upd.html); some of these cases have been considered to exhibit homozygous expression of a recessive mutation (**Table 2**) (Brzustowicz et al., 1994; Seal et al., 2006; Lin et al., 2007; Garcia et al., 2014; Melone et al., 2014; Numata et al., 2014; Janecke et al., 2015; Wang et al., 2015). Imprinting effects due to uniparental disomy of chromosome 5 are possible, but this has not been established. Further studies on uniparental disomy of chromosome 5 are necessary to clarify the prevalence and possibility of imprinting defects.

**Table 2:** Uniparental disomy of chromosome 5 related to autosomal recessive disorders.

	Case 1	Case 2	Case 3	Case 4	Case 5	Case 6	Case 7	Our case
Reference	Brzustowiczet al. (1994)	Seal et al. (2006)	Numata et al. (2014)	Garcia et al. (2014)	Melone et at. (2014)	Janecke et al. (2015)	Wang et al. (2015)	Park et al. (2019)
Phenotype	Spinal muscular atrophy	Child-onset schizophrenia	Netherton syndrome	Multiple epiphyseal dysplasia	Stüve-Wiedemann syndrome	Congenital sodium diarrhea	Laron dwarfism	hereditary sensory and autonomic neuropathy 2B
Origin	Paternal	Paternal	Maternal	Paternal	Maternal	Maternal	Undetermined	Paternal
Complete/Segmental isodisomy	Complete isodisomy	Segmental isodisomy (5q32-qter)	Complete isodisomy	Complete isodisomy	Complete isodisomy	Complete isodisomy	Undetermined	Complete isodisomy
Caused gene or Chrosomosome loci	considered *SMN*	Undetermined	*SPINK5*	*SLC26A2*	*LIFR*	*SLC9A3*	*GHR*	*RETREG1*
Variant			c.1732C > T(p.Arg578X)	C.835C > T(p.Arg279Trp)	C.2170C > G(p.Pro724Ala)			c.765dupT(p.Gly256TrpfsTer7)

We reported the case of a patient born to non-consanguineous parents who was diagnosed with HSAN 2B caused due to a novel frameshift mutation (c.765dupT/p.Gly256TrpfsTer7) in the *RETREG1* gene and paternal uniparental isodisomy of chromosome 5. Clinicians should be aware that autosomal recessive disorders due to homozygous variants can occur because of uniparental disomy in offspring of non-consanguineous parents.

## Data Availability Statement

The raw data supporting the conclusions of this manuscript will be made available by the authors, without undue reservation, to any qualified researcher.

## Ethics Statement

This study was carried out in accordance with the recommendations of the institutional research review board of the Catholic University of Korea, Incheon St. Mary’s Hospital with written informed consent from all subjects. All subjects gave written informed consent in accordance with the Declaration of Helsinki. The protocol was approved by the institutional research review board of the Catholic University of Korea, Incheon St. Mary’s Hospital.

## Author Contributions

D-WL, J-HJ, and JJ contributed to prepare the case. G-YP wrote the article and critically reviewed article. D-HJ takes responsibility for the study as a whole.

## Funding

This work was supported by the National Research Foundation of Korea (NRF) grant funded by the Korea government (MSIT) (No. 2017R1C1B5014840). This research was supported by a Grant of Translational R&D Project through Clinical Research Laboratory, Incheon St. Mary’s Hospital.

## Conflict of Interest

Author JJ was employed by company EONE-Diagnomics Genome Center, South Korea. Author J-HJ was employed by company Green Cross Genome, South Korea. The remaining authors declare that the research was conducted in the absence of any commercial or financial relationships that could be construed as a potential conflict of interest.

## Abbreviations

HSAN, Hereditary Sensory and Autonomic Neuropathy.
